# Protein phosphatase 2A-B55δ enhances chemotherapy sensitivity of human hepatocellular carcinoma under the regulation of microRNA-133b

**DOI:** 10.1186/s13046-016-0341-z

**Published:** 2016-04-14

**Authors:** Qunying Zhuang, Tengjian Zhou, Chengyong He, Shili Zhang, Yang Qiu, Bing Luo, Ran Zhao, Hengchuan Liu, Yuchun Lin, Zhongning Lin

**Affiliations:** State Key Laboratory of Molecular Vaccinology and Molecular Diagnostics, School of Public Health, Xiamen University, Xiang’an South Rd., Xiamen, Fujian 361102 PR China

**Keywords:** PP2A, B55δ, Hepatocellular carcinoma, Chemotherapy sensitivity, microRNA-133b, Cisplatin

## Abstract

**Background:**

Hepatocellular carcinoma (HCC) remains a major public health problem worldwide. The identification of effective chemotherapeutic targets for advanced HCC patients is urgently required. In this study, we investigated the role of protein phosphatase 2A-B55δ subunit (PP2A-B55δ, encoded by the *PPP2R2D* gene) and related mechanisms affecting chemotherapy sensitivity of HCC.

**Methods:**

Experimental approaches for measuring the levels of *PPP2R2D* mRNA and B55δ protein in HCC included bioinformatics analyses, quantitative real-time polymerase chain reaction (qRT-PCR), western blotting (WB), immunofluorescence and immunohistochemistry assays. Cell cycle, migration, colony formation, apoptosis, and cell proliferation assays in stable *PPP2R2D*-knockdown and -overexpression cell lines *in vitro*, and tumorigenicity assays *in vivo*, were performed to explore the function of B55δ in cisplatin (cDDP) chemotherapy of HCC. Bioinformatics prediction, luciferase reporter assays, qRT-PCR, WB, and cell cycle analyses were used to reveal the regulatory relationship between microRNA-133b (miR-133b) and *PPP2R2D* expression. miR-133b mimic and inhibitor were used to elucidate the regulatory mechanism.

**Results:**

Our studies showed that *PPP2R2D* expression was down-regulated in both HCC tumors and HCC cell lines. Treatment with cDDP increased the amount of B55δ protein. Artificially increasing the expression of B55δ counteracted cyclin-dependent kinase 1 activation, modulated transitions of the cell cycle, and increased the suppressive effect of cDDP on cell migration, colony formation, apoptosis, and proliferation *in vitro* and tumor growth *in vivo*, thus enhancing therapeutic efficiency. In contrast, knockdown of B55δ partially inhibited the effect of cDDP chemotherapy. miR-133b was shown to regulate *PPP2R2D* expression by binding to the 3’-untranslated region of *PPP2R2D* mRNA. The miR-133b/*PPP2R2D* signaling pathway affects the effectiveness of cDDP chemotherapy.

**Conclusions:**

PP2A-B55δ, regulated by miR-133b, enhances the sensitivity of HCC to cDDP chemotherapy. Our data indicate that PP2A-B55δ might be a novel and attractive target for increasing chemotherapy sensitivity of HCC.

**Electronic supplementary material:**

The online version of this article (doi:10.1186/s13046-016-0341-z) contains supplementary material, which is available to authorized users.

## Background

According to global cancer statistics, about 782,500 new liver cancer cases occurred worldwide in 2012, together with about 745,500 deaths [1]. Seventy to 90 % of primary liver cancers are hepatocellular carcinoma (HCC). Since patients are asymptomatic at the early stages of the disease, HCC is mostly diagnosed at an advanced stage [2]. Moreover, the effectiveness of anti-cancer treatment varies considerably among different HCC patients. Therefore, it is necessary to identify targets for enhancing the sensitivity to chemotherapeutic management of HCC.

In recent years, a growing number of studies have shown that protein phosphatase 2A (PP2A) is an important tumor suppressor. As a crucial member of the serine/threonine protein phosphatase family widely expressed in eukaryotic cells, PP2A is involved in the regulation of the signal transduction, cell cycle, proliferation, differentiation, apoptosis, and other processes [3]. PP2A-B55δ subunit, encoded by the *PPP2R2D* gene, is one of four isoforms (α, β, γ, and δ) of the PP2A B55 regulatory subunit family [4]. The interaction between B55δ and cyclin-dependent kinase 1 (CDK1) is reported to play a critical role in cell cycle progression [5]. However, it is still unclear whether B55δ enhances chemotherapy sensitivity of HCC cells by regulating the cell cycle.

MicroRNAs (miRNAs) are small non-coding RNAs that regulate gene expression either through mRNA degradation or translational repression [6]. The interaction with the 3’-untranslated region (3’UTR) of the targeted mRNAs via base pairing is thought to be the main mechanism of miRNA function [7]. As nodes of signaling networks, miRNAs play a role in the regulation of metabolic homeostasis and cancer development [8–10]. Recent studies suggest a number of clinically significant miRNAs that may target PP2A [11]. In view of the lack of conclusive information on the miRNA regulation of *PPP2R2D*, this study aimed to characterize the functional role of miRNA targeting of *PPP2R2D* in the chemotherapy of HCC*.*

As a key drug in the treatment of advanced HCC [12], cisplatin (cDDP) was selected as a representative chemotherapeutic drug of HCC in this study. We explored the role of PP2A-B55δ both in regulating the cell cycle, migration, colony formation, apoptosis, and proliferation of human hepatoblastoma HepG2 cells and in tumor growth in xenograft mice in the presence of cDDP, and we investigated the details of the microRNA-133b (miR-133b)/*PPP2R2D* signaling pathway. We concluded that PP2A-B55δ, under the regulation of miR-133b, could serve as a promising target for increasing chemotherapy sensitivity of HCC.

## Methods

### Bioinformatics analysis

Gene expression data of HCC cohorts were acquired from the Gene Expression Omnibus (GEO) database (http://www.ncbi.nlm.nih.gov/geo/). The public databases microRNA.org (http://www.microrna.org/microrna/getGeneForm.do) and TargetScan (http://www.targetscan.org/vert_70/) were used to screen for miRNAs which might target *PPP2R2D*. The predicted free energies of the binding between miRNAs and their targets were calculated by the RNAhybrid program (http://bibiserv.techfak.uni-bielefeld.de/rnahybrid/).

### Cell culture and reagents

The human hepatic cell line L02, HCC cell lines HepG2, MHCC97H, MHCC97L, Hep3B, Huh7 and human embryonic kidney 293T cells (HEK-293T) were obtained from the Cancer Center of Sun Yat-sen University (Guangzhou, China). The human hepatic cell line QSG7701 and HCC cell line QGY7703 were obtained from the College of Chemistry and Chemical Engineering, Xiamen University (Xiamen, China). L02, QSG7701, HepG2, Hep3B, and QGY7703 were cultured in RPMI-1640 medium (Gibco, NY, USA) supplemented with 10 % fetal bovine serum (FBS, Gibco) and 1 % penicillin-streptomycin (P&S, Gibco). MHCC97H, MHCC97L, and Huh7 were cultured in DMEM (Gibco) with 10 % FBS and 1 % P&S. All cell lines were maintained at 37 °C in a 5 % CO_2_ humidified incubator (Thermo, CO, USA). cDDP was obtained from Hansoh Pharmaceutical Co., Ltd (Jiangsu, China).

### Antibodies

The primary antibodies for PP2A-B55δ were purchased from Abcam (MA, USA) and Santa Cruz (CA, USA). PP2A-Aα subunit antibody was from Covance (NJ, USA). PP2A-C subunit, phosphorylated CDK1 (p-CDK1 Tyr15), CDK1, and cleaved Caspase-3 antibodies, and the secondary antibodies anti-rabbit IgG and anti-mouse IgG were from Cell Signaling Technology (MA, USA). PP2A-B55α antibody was from Merck Millipore (MA, USA). PP2A-B56γ antibody was obtained from Thermo. Glyceraldehyde-3-phosphate dehydrogenase (GAPDH) and B-cell lymphoma 2-related protein (Bcl-2) antibodies were from Beyotime (Shanghai, China). Antibodies for Cyclin B1, Cyclin E1, Bcl-2-associated X protein (Bax), and proliferating cell nuclear antigen (PCNA) were from Ruiying Bioalogical (Jiangsu, China). The secondary antibodies anti-rat IgG and anti-goat IgG were from Proteintech (IL, USA). The fluorescein isothiocyanate-conjugated (FITC) rabbit anti-goat IgG was from Liankebio (Zhejiang, China).

### Quantitative real-time polymerase chain reaction (qRT-PCR)

Total RNA was extracted using TRIzol® Reagent (Ambion, TX, USA) and reverse-transcribed into cDNA using PrimeScript™ RT reagent kit (TaKaRa, Otsu, Japan). qRT-PCR was performed with SYBR® *Premix Ex Taq*™ II kit (TaKaRa) in a CFX96 Touch™ Real-Time PCR Detection System (Bio-Rad, CA, USA) using the primers (Sangon Biotech, Shanghai, China) shown in Additional file 1: Table S1. Cycling conditions were 95 °C for 30 s, 40 cycles of 95 °C for 5 s, and 60 °C for 34 s. The mRNA levels of genes were evaluated using the 2^-△△Ct^ relative quantification method. *ACTB* (encoding β-actin) was used as a reference control. Quantitative analysis of miRNA expression was performed with the Bulge-Loop™ hsa-miR-133b qRT-PCR primer set (Ribobio, Guangzhou, China). U6 snRNA was used as a reference control.

### Western blotting (WB) analysis

Cells were lysed in whole-cell lysate buffer. For phosphorylated protein, 1 % phosphatase inhibitor cocktail was added to the whole-cell lysate buffer. Protein lysates were resolved by 10 % or 12 % sodium dodecyl sulfate-polyacrylamide gel electrophoresis (SDS-PAGE), and then transferred to poly vinylidene fluoride (PVDF) membranes (Pall, NY, USA). After blocking with 5 % nonfat milk, the membranes were incubated with primary antibodies overnight at 4 °C, and then incubated with the corresponding secondary antibodies at room temperature for 1 h. Protein bands were visualized with an enhanced chemiluminescence kit (Pierce, IL, USA). The blot intensities of each band were analyzed by ImageJ software (NIH, MD, USA). GAPDH was used as a loading control.

### PP2A activity assay

A Serine/Threonine Phosphatase Assay System (Promega, WI, USA) was used for measuring PP2A activities. Following the instruction manual, collected cell lysates were centrifuged at 1 × 10^5^ *g* for 1 h at 4 °C in phosphatase storage buffer. Sephadex® G-25 spin columns were used to remove endogenous phosphate. The treated lysates were added to a mixture containing PP2A reaction buffer and phosphopeptide, and then incubated for 1 h at 37 °C. The reaction was stopped with molybdate dye/additive mixture. The optical density (OD) of the samples was read using a Multiskan™ FC microplate photometer (Thermo) at 600 nm. PP2A activity was measured in three parallel experiments.

### Immunofluorescence assay

The cells, seeded on coverslips in 12-well plates, were fixed with freshly-prepared 4 % paraformaldehyde and permeabilized with 0.5 % Triton X-100. After blocking with phosphate-buffered saline (PBS) containing 1 % BSA, the cells were treated with primary antibody overnight and incubated with fluorescent secondary antibody for 1 h in the dark. After extensive washing, cell nuclei were counterstained with 4, 6-diamidino-2-phenylindole (DAPI) for 1 min. The cells were photographed using a confocal microscope (Olympus, Tokyo, Japan). Three independent assays were conducted; representative images are shown.

### Establishment of stable *PPP2R2D*-knockdown cell lines

The short hairpin RNA (shRNA) targeting the *PPP2R2D* mRNA sequence (GenBank Accession No. NM_018461.4) was designed using the Genetic Perturbation Platform (http://www.broadinstitute.org/rnai/public/). The sense and antisense oligonucleotides of sh*2R2D* (Additional file 1: Table S1) were annealed and then ligated into lentiviral vector pLKO.1-puro (a kind gift from Dr. Wen Chen, Sun Yat-sen University, China) to construct the pLKO.1-sh*2R2D* recombinant plasmid. The corresponding control plasmid was pLKO.1 bearing shRNA targeting green fluorescent protein (pLKO.1-sh*GFP*) [13]. The lentiviral plasmid (pLKO.1-sh*GFP* or pLKO.1-sh*2R2D*), packaging plasmid (pCMV-delta 8.9), and envelope plasmid (VSVG) were co-transfected into HEK-293T cells using X-tremeGENE HP DNA transfection reagent (Roche, IN, USA). Lentiviruses packaged in HEK-293T cells were purified and then introduced into HepG2 cells using polybrene (Sigma, MO, USA). The established cell lines (HepG2-sh*GFP* and HepG2-sh*2R2D*) were screened with 0.6 μg/ml puromycin.

### Establishment of stable *PPP2R2D*-overexpression cell lines

*PPP2R2D* full-length coding sequence (*2R2Dc*) was obtained using the primers shown in Additional file 1: Table S1 and incorporated into retroviral vector pBabe-puro (a kind gift from Dr. Wen Chen) to construct the pBabe-*2R2Dc* recombinant plasmid. HEK-293T cells were co-transfected with retroviral plasmid (pBabe or pBabe-*2R2Dc*) and pCL-Ampho vector in a ratio of 1:1. HepG2 cells were then infected with retroviruses produced by HEK-293T cells. The established cell lines (HepG2-pBabe and HepG2-*2R2Dc*) were screened with 0.6 μg/ml puromycin.

### Cell cycle analysis

Cells were collected, fixed in 70 % ice-cold ethanol, incubated with RNase, and dyed with propidium iodide (PI). Flow cytometry (FCM, Beckman, CA, USA) was used to analyze DNA content. The data were analyzed using ModFit LT 3.1 (Verity Software House, ME, USA) and cell cycle distribution was calculated. Each experiment was repeated at least three times.

### Cell migration assay

The migratory ability of cells was evaluated using 6.5 mm Transwell chambers with 8.0 μm pore polycarbonate membrane insert (Corning, NY, USA). Cells prepared in FBS-free medium were seeded onto the upper chambers, and medium with 10 % FBS was added to the bottom chambers as a chemoattractant. cDDP was given to the cells 30 min later. After 12 h, migrated cells located on the lower surface of chambers were fixed and stained with crystal violet. The cells were photographed using an inverted microscope and counted in 10 randomly selected fields at a 200× magnification using Image Pro-Plus software 6.0 (IPP 6.0, Media Cybernetics, MD, USA). The assay was carried out three times.

### Cell colony formation assay

To evaluate the self-renewal capacity of different cell types, 800 cells were seeded in 6-well plates. After 24 h, cells were treated with cDDP for 12 h and maintained in culture for another 6 days. On day 7, the cells were fixed, stained, and drained for taking photographs. The colony formation areas were calculated by ImageJ. The assay was carried out in triplicate.

### Apoptosis assay

The Annexin V‑FITC Apoptosis Detection kit (Beyotime) was used for apoptosis assays. Briefly, cells were harvested, resuspended in Annexin V-FITC binding buffer, and stained with Annexin V-FITC and PI in the dark. Cells were then analyzed by FCM. FlowJo v.7.6.5 (FlowJo, OR, USA) was used to analyze the data. The experiment was repeated three times separately.

### Cell proliferation assay

Cells were plated at a density of 8 × 10^3^ cells/well in 96-well plates and exposed to cDDP for 24 h. Then, 3-(4, 5-dimethylthiazol-2-yl)-2, 5-diphenyltetrazolium bromide (MTT, Sigma) was added to each well. The OD was measured spectrophotometrically at 570 nm using a Multiskan™ FC microplate photometer 4 h later. The cell growth inhibition (GI) rate was calculated as the following formula: GI (%) = [1 − (OD_570Sample_ − OD_570Blank_)/(OD_570Ctrl_ − OD_570Blank_)] × 100 %. This assay was repeated at least three times.

### *In vivo* xenograft studies

BALB/c nude mice (5–7 weeks old) were obtained from the Xiamen University Laboratory Animal Center (Xiamen, China). All experimental procedures were approved by the Experimental Animal Ethics Committee of Xiamen University. The mice were randomly allocated to four groups: pBabe-Ctrl group, *2R2Dc*-Ctrl group, pBabe-cDDP group, and *2R2Dc*-cDDP group (*n* = 6 in each group). A total of 5 × 10^6^ cells (HepG2-pBabe or HepG2-*2R2Dc*) were suspended in PBS with matrigel (BD Biosciences, CA, USA) in a 1:1 ratio. These cell suspensions were subcutaneously inoculated into the right flank of each mouse, and the tumors were allowed to grow for a week. Tumor width (w) and length (l) and body weights were measured every other day. Tumor volume (V) was calculated using the formula: V = (w^2^ × l)/2. A week later, the mice in the *2R2Dc*-cDDP group or the pBabe-cDDP group were injected with 1 mg/kg body weight cDDP via tail vein every other day for three times, respectively. All mice were sacrificed and tumors were excised on day 14. Tumor weight was recorded, and tumor tissues were subjected to immunohistochemistry (IHC), WB, and qRT-PCR assays.

### Immunohistochemistry (IHC)

The dissected tumor tissues were fixed, embedded in paraffin, and serially sectioned at 4 μm thickness. Sections were dewaxed, rehydrated, and antigen retrieved. UltraSensitive™ SP (Mouse/Rabbit) IHC Kit (Maixin Biotech, Fuzhou, China) was used according to the manufacturer’s instructions. Sections were incubated with primary antibodies overnight followed by a secondary antibody. Next, sections were stained with 3, 3’-diaminobenzidine, counterstained with hematoxylin, and dehydrated through graded ethanol solutions. Finally, sections were photographed using a microscope and analyzed using IPP 6.0.

### Transient transfection and luciferase reporter assay

The *PPP2R2D* 3’UTR fragment was amplified using the primers shown in Additional file 1: Table S1, ligated into the pGL3-control luciferase reporter vector (pGL3c, a kind gift from Dr. Shimei Zhuang, Sun Yat-sen University, China), and confirmed by sequencing. Transfection was performed in 96-well plates using Lipofectamine® 2000 transfection reagent (Invitrogen, CA, USA) according to the standard protocol. Briefly, cells were co-transfected with 50 ng pRL-TK vector and 200 ng of either pGL3c or pGL3c-*2R2D*-3’UTR for 4 h. After treatment with cDDP for 6 h, cells were analyzed using Dual Luciferase® reporter assay system (Promega). The luciferase activities were normalized against the activity of pGL3c.

### miRNA mimic or inhibitor transfection

Cells were transfected with miRNA mimic or inhibitor (Ribobio) according to the manufacturer’s instructions. Briefly, cells were plated in 6-well plates and grown to 70 % confluence. For each well, 50 nM miR-133b mimic or 100 nM inhibitor in ribo*FECT*™ CP Buffer (Ribobio) was mixed gently with ribo*FECT*™ CP Reagent and added to the cells for 12 h.

### Statistics

Statistical analyses were carried out using the Statistical Package for Social Sciences (SPSS) version 16.0 (SPSS, IL, USA). All data were expressed as mean ± standard deviation (SD). The statistical significance was determined using two-tailed unpaired Student’s *t-*test for the comparison of two groups and using one-way analysis of variance (ANOVA) for the comparison of multiple groups, following which the statistical significance was determined by Dunnett’s *t*-test. Correlation between variables was evaluated with Pearson’s correlation for normal distribution or Spearman’s correlation for non-normal distribution. A *P* < 0.05 was considered statistically significant.

## Results

### *PPP2R2D* is down-regulated in HCC

**Fig. 1:**
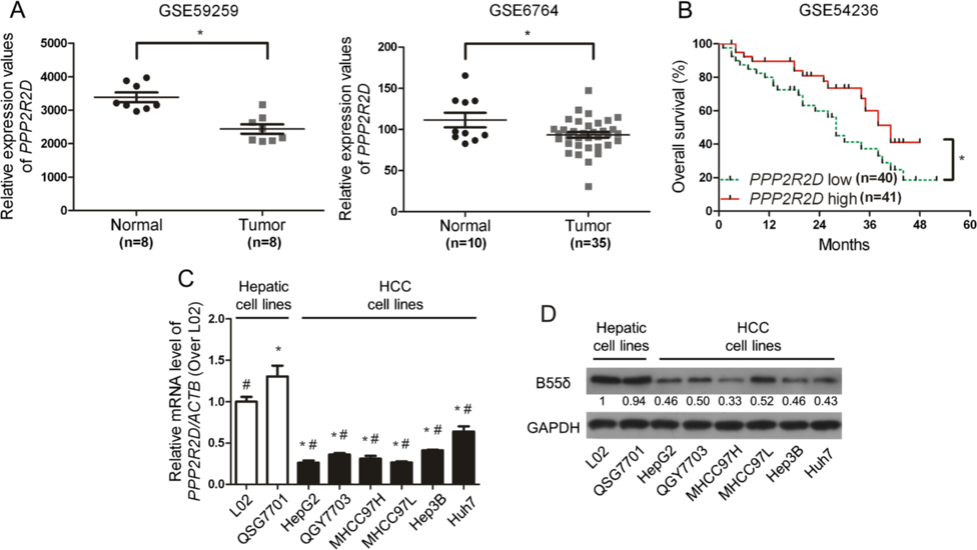
*PPP2R2D* is down-regulated in HCC. **a** *PPP2R2D* expression levels in two cohorts of HCC patients [14, 15]. The mRNA microarray data were obtained from GEO database (Accession No.: GSE59259 and GSE6764, *P < 0.05). **b** Kaplan-Meier analysis showing overall survival of HCC patients [16] with two different levels of *PPP2R2D* expression (Accession No.: GSE54236, *PPP2R2D* expression was treated as a binary variable divided into high or low expression according to the median, Log-rank, *P < 0.05). **c** *PPP2R2D* mRNA and (**d**) B55δ protein levels were evaluated in normal hepatic cell lines and HCC cell lines. The data shown in the bar graph are the mean ± SD of three independent experiments. *P < 0.01 as compared with L02 cells; ^#^ *P* < 0.01 as compared with QSG7701 cells

In order to investigate the association between *PPP2R2D* expression and HCC, we analyzed the published microarray datasets available from GEO database. In two different HCC cohorts (Accession No.: GSE59259 and GSE6764) [14, 15], the tumor tissues exhibited relatively low *PPP2R2D* expression levels (Fig. 1a). In addition, Kaplan-Meier survival curves shown in Fig. 1b indicated that the 5-year overall survival rates of HCC patients (Accession No.: GSE54236) [16] with low *PPP2R2D* expression was lower than that with high *PPP2R2D* expression.

We further evaluate *PPP2R2D* mRNA and B55δ protein in two normal hepatic cell lines and six HCC cell lines by qRT-PCR and WB. The data showed that the levels of *PPP2R2D* mRNA and B55δ protein were significantly suppressed in all HCC cell lines compared with normal hepatic cell lines (Fig. 1c–d). Taken together, the expression of *PPP2R2D* was down-regulated in HCC tissues and cells, indicating that *PPP2R2D* might act as a tumor suppressor in the chemotherapy of HCC.

### The expression of B55δ is increased by cDDP in HepG2 cells

**Fig. 2:**
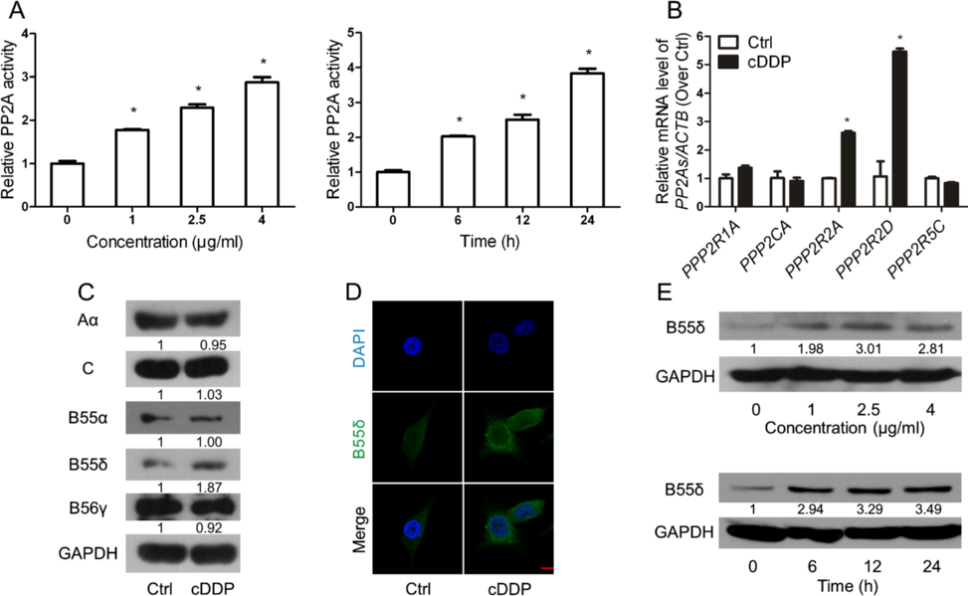
The expression of B55δ is increased by cDDP in HepG2 cells. **a** PP2A activity and (**e**) B55δ protein levels in HepG2 cells treated with 0, 1, 2.5, or 4 μg/ml cDDP for 12 h, or treated with 2.5 μg/ml cDDP for 0, 6, 12, or 24 h. **b**-**d** HepG2 cells were treated with or without 2.5 μg/ml cDDP for 12 h. **b** The mRNA levels of PP2A subunits (*PPP2R1A*, *PPP2CA*, *PPP2R2A*, *PPP2R2D* and *PPP2R5C*). **c** The protein levels of PP2A-Aα, -C, -B55α, -B55δ, and -B56γ subunits. **d** Immunofluorescence images of B55δ in HepG2 cells (×630 magnification; scale bar, 10 μm). Data are presented as mean ± SD of three independent assays. *P < 0.01 as compared with cDDP-untreated group (Ctrl)

In order to explore the functional role of *PPP2R2D* in the chemotherapy of HCC, HepG2 cells were chosen as a sensitive model because of its lower expression of *PPP2R2D* compared to other HCC cell lines (Fig. 1c–d). We used cDDP, an accepted anti-tumor agent [17], investigating B55δ expression in HepG2 cells in response to chemotherapy. PP2A activity was increased by cDDP in a concentration- and time-dependent manner (Fig. 2a). As shown in Fig. 2b, cDDP significantly up-regulated the mRNA level of *PPP2R2D* and *PPP2R2A*, but not of *PPP2R1A*, *PPP2CA*, or *PPP2R5C*. cDDP increased only the expression of B55δ protein, not of the other subunits of PP2A (Fig. 2c). Immunofluorescence analysis also revealed that B55δ was more strongly expressed following cDDP treatment (Fig. 2d). Additionally, the protein levels of B55δ were increased in cDDP-treated cells in a concentration- and time-dependent manner (Fig. 2e). Thus, our findings suggested that cDDP increased the amount of B55δ in HepG2 cells.

### Knockdown of *PPP2R2D* partially reverses the sensitivity of HepG2 cells to cDDP

**Fig. 3:**
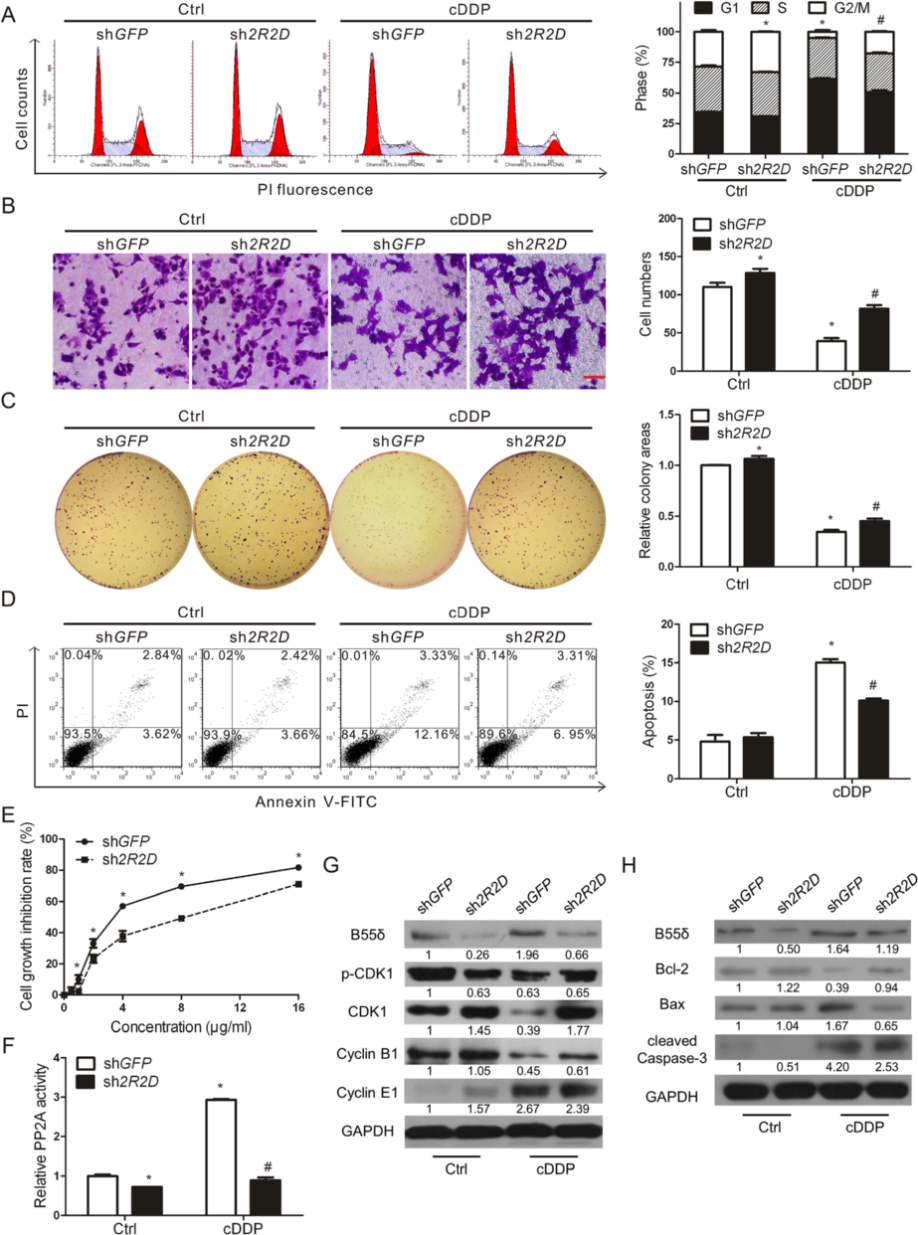
B55δ knockdown partially reverses the sensitivity of HepG2 cells to cDDP. HepG2-sh*GFP* and HepG2-sh*2R2D* cells were treated with or without 2.5 μg/ml cDDP for 12 h. **a** Cell cycle distribution was analyzed by FCM. **b** Cell migratory ability was detected by transwell migration assay. The migrated cells were photographed and counted (×200 magnification; scale bar, 100 μm). **c** Cell self-renewal capacity was measured by colony formation assay. **d** Cell apoptosis was detected by Annexin V-FITC assay and analyzed by FCM. **e** Cell growth inhibition rate of the two cells treated with 0–16 μg/ml cDDP for 24 h was analyzed using cell proliferation assay. **f** PP2A activity was determined by the Serine/Threonine Phosphatase Assay System. **g**-**h** The expression of related proteins B55δ, p-CDK1, CDK1, Cyclin B1, Cyclin E1, Bcl-2, Bax, and cleaved Caspase-3 was determined by WB assays. Data shown are the mean ± SD of three independent assays. *P < 0.01 as compared with Ctrl group of HepG2-sh*GFP* cells; ^#^ *P* < 0.01 as compared with cDDP group of HepG2-sh*GFP* cells

To elucidate the crucial role that B55δ plays in sensitizing HepG2 cells to anti-cancer treatment, *PPP2R2D* was knocked down. Approximately 50 % knockdown of *PPP2R2D* mRNA level was achieved in HepG2-sh*2R2D* cells as compared with HepG2-sh*GFP* cells, which resulted in a 2-fold decrease of B55δ protein without alteration of other PP2A subunits (Additional file 2: Figure S1A–B). G1 phase cell numbers were mildly decreased by sh*2R2D* (“Ctrl” in Fig. 3a). The migratory ability and self-renewal capacity of HepG2-sh*2R2D* cells were both higher than those of HepG2-sh*GFP* cells (“Ctrl” in Fig. 3b–c). There was no apparent difference between two established stable cell lines in apoptosis rate (“Ctrl” in Fig. 3d). However, knockdown of *PPP2R2D* significantly reversed G1 phase cell cycle arrest, migratory inhibition, cell colony area reduction, and apoptosis induced by cDDP (Fig. 3a–d). After treatment with cDDP, cell growth inhibition rate of HepG2-sh*2R2D* cells was also markedly lower than that of HepG2-sh*GFP* cells (Fig. 3e). In addition, knockdown of *PPP2R2D* significantly decreased the PP2A activity after treatment with cDDP (Fig. 3f), which correspondingly influenced the levels of p-CDK1 and cell cycle-related proteins Cyclin B1 and Cyclin E1 (Fig. 3g). The apoptosis-related proteins (Bcl-2, Bax, and cleaved Caspase-3) were in perfect agreement with the FCM results (Fig. 3h). Based on these results, we concluded that knockdown of *PPP2R2D* partially reversed the sensitivity of HepG2 cells to cDDP.

### Overexpression of B55δ sensitizes HepG2 cells to cDDP

**Fig. 4:**
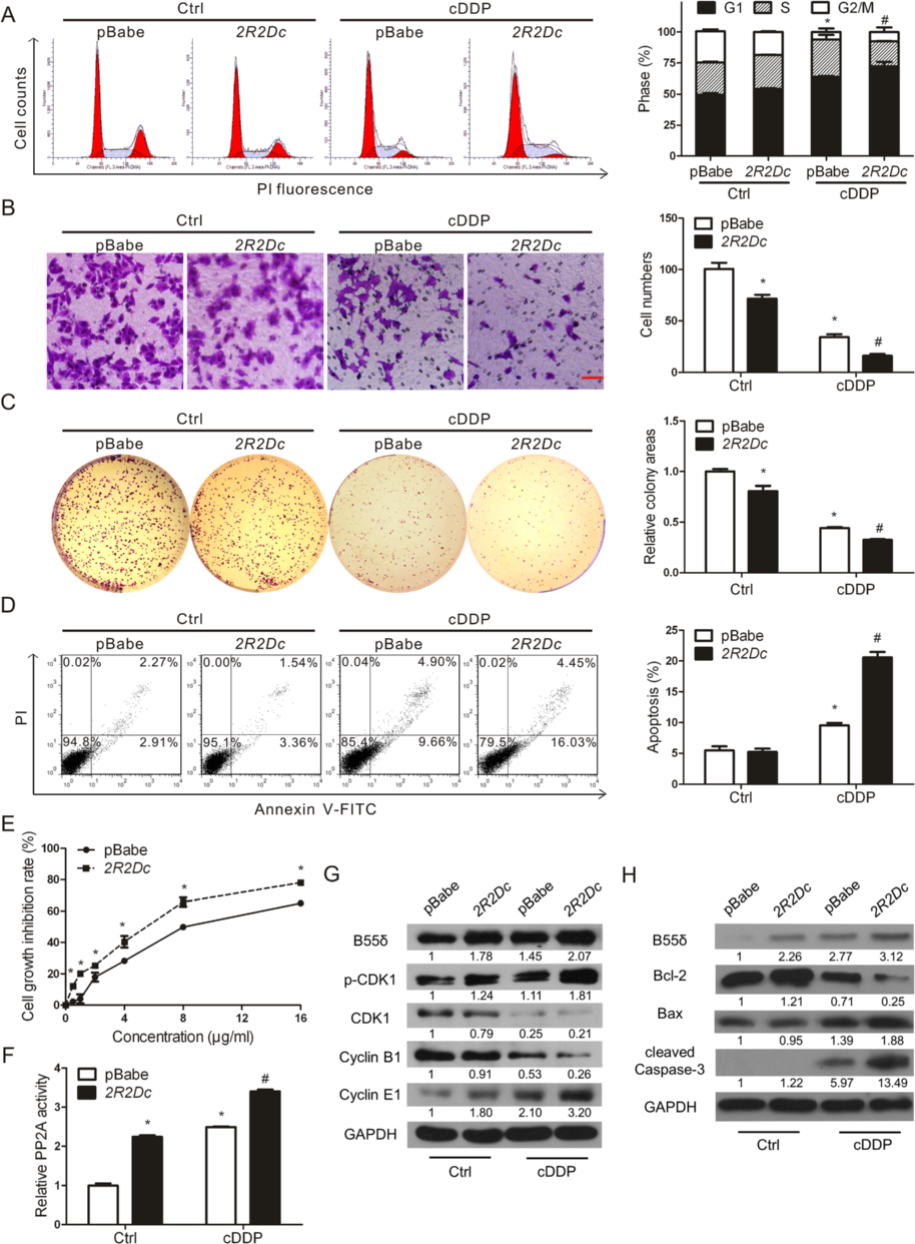
Overexpression of B55δ sensitizes HepG2 cells to cDDP. HepG2-pBabe and HepG2-*2R2Dc* cells were treated with or without 2.5 μg/ml cDDP for 12 h. **a** Cell cycle distribution, (**b**) migratory ability, (**c**) self-renewal capacity, (**d**) cell apoptosis, and (**f**) PP2A activity were detected. **e** Cell growth inhibition rate of the two cells treated with 0-16 μg/ml cDDP for 24 h was analyzed. **g**-**h** Expression of B55δ, p-CDK1, CDK1, Cyclin B1, Cyclin E1, Bcl-2, Bax, and cleaved Caspase-3 was measured. Values shown are mean ± SD of three independent experiments. * *P* < 0.01 as compared with Ctrl group of HepG2-pBabe cells; ^#^ *P* < 0.01 as compared with cDDP group of HepG2-pBabe cells

We further established stable cell lines of HepG2 overexpressing B55δ and confirmed the overexpression at both the mRNA and protein levels (Additional file 2: Figure S1C–D). G1 phase cell numbers of HepG2-*2R2Dc* cells were a little more than those of HepG2-pBabe cells (“Ctrl” in Fig. 4a). The migratory ability and self-renewal capacity were inhibited by overexpression of B55δ (“Ctrl” in Fig. 4b–c). Also, there was no apparent difference between apoptosis rates of the two stable cell lines (“Ctrl” in Fig. 4d). Overexpression of B55δ remarkably exacerbated G1 phase arrest, migratory inhibition, colony area reduction, and apoptosis induced by cDDP (Fig. 4a–d). In addition, the proliferation of HepG2-*2R2Dc* cells was inhibited more than that of HepG2-pBabe cells after treatment with cDDP (Fig. 4e). As shown in Fig. 4f, the level of PP2A activity in HepG2-*2R2Dc* cells with cDDP treatment was higher than that in HepG2-pBabe cells, which correspondingly influenced the phosphorylation level of CDK1 (“p-CDK1” in Fig. 4g). The expression levels of cell cycle-related proteins and apoptosis-related proteins were consistent with FCM analysis (Fig. 4g–h). To sum up, overexpression of B55δ sensitized HepG2 cells to cDDP, so B55δ seems to play an important role in the chemotherapy of HCC.

### Overexpression of B55δ promotes the sensitivity of HCC xenograft tumors to cDDP

**Fig. 5:**
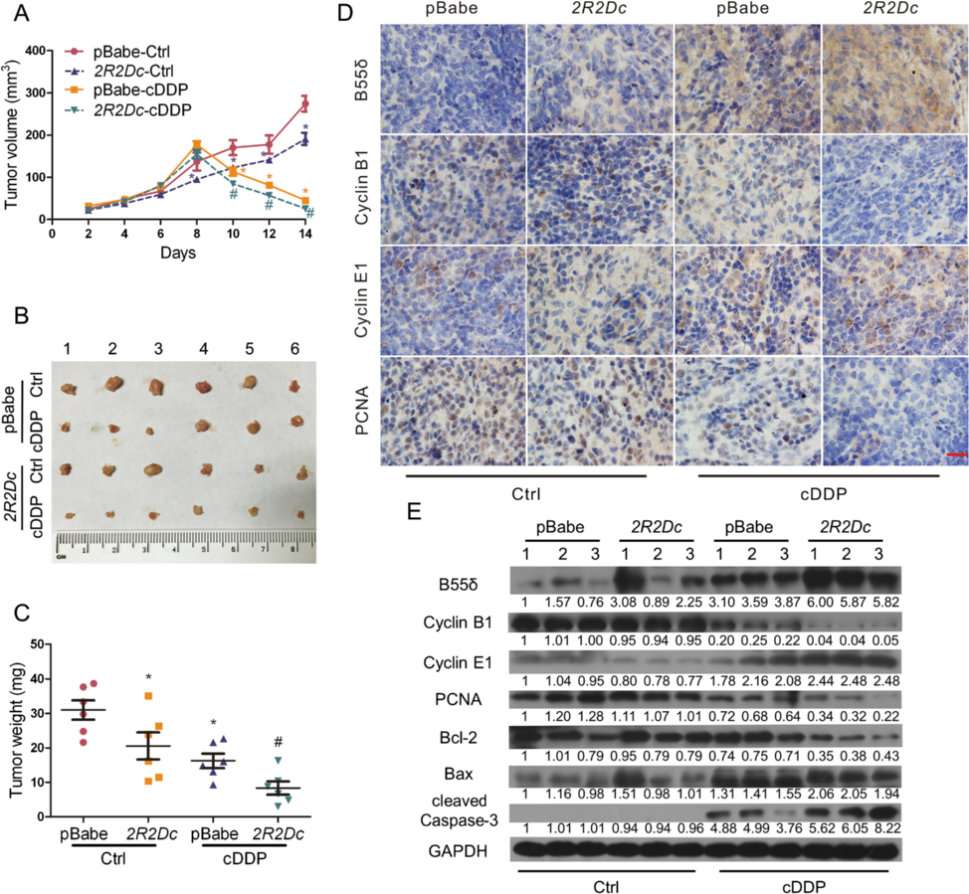
Overexpression of B55δ promotes the sensitivity of HCC xenograft tumors to cDDP. Nude mice transplanted with HepG2-pBabe or HepG2-*2R2Dc* cells were injected with or without 1 mg/kg body weight cDDP every other day for 3 times. **a** Graph depicts the tumor volume of the four groups (pBabe-Ctrl, pBabe-cDDP, *2R2Dc*-Ctrl, and *2R2Dc*-cDDP). **b** Representative images of tumors excised from nude mice. **c** Scatter plot shows the tumor weights for the four groups. Values shown are mean ± SD of six different samples. *P < 0.01 as compared with pBabe-Ctrl group; ^#^ *P* < 0.01 as compared with pBabe-cDDP group. **d** IHC images of sections of tumors reveal the expression of B55δ, Cyclin B1, Cyclin E1, and PCNA (×1000 magnification; scale bar, 20 μm). **e** WB was performed to detect B55δ, Cyclin B1, Cyclin E1, PCNA, Bcl-2, Bax, and cleaved Caspase-3 protein levels in 12 representative tumor tissues (*n* = 3 in each group)

To confirm the tumor suppressive function of B55δ *in vivo*, tumorigenicity assays were performed. The data showed that B55δ overexpression reduced xenograft tumor growth (Fig. 5a). As shown in Fig. 5a–c, tumor volume, size, and weight of the *2R2Dc*-cDDP group were significantly smaller than those of the pBabe-cDDP group. B55δ was up-regulated in the xenograft tumor tissues from the *2R2Dc*-cDDP group (Fig. 5d–e). Furthermore, there was a down-regulation of Cyclin B1 and PCNA, and an up-regulation of Cyclin E1 in the *2R2Dc*-cDDP group (Fig. 5d–e, Additional file 3: Figure S2). Figure 5e shows higher expression levels of Bax and cleaved Caspase-3 and a lower Bcl-2 level in the *2R2Dc*-cDDP group. Taken together, *in vivo* data supported *in vitro* findings indicating that overexpression of B55δ increased the therapeutic susceptibility of HCC xenograft tumors to cDDP.

### miR-133b regulates the expression of *PPP2R2D* by binding to its 3’UTR

**Fig. 6:**
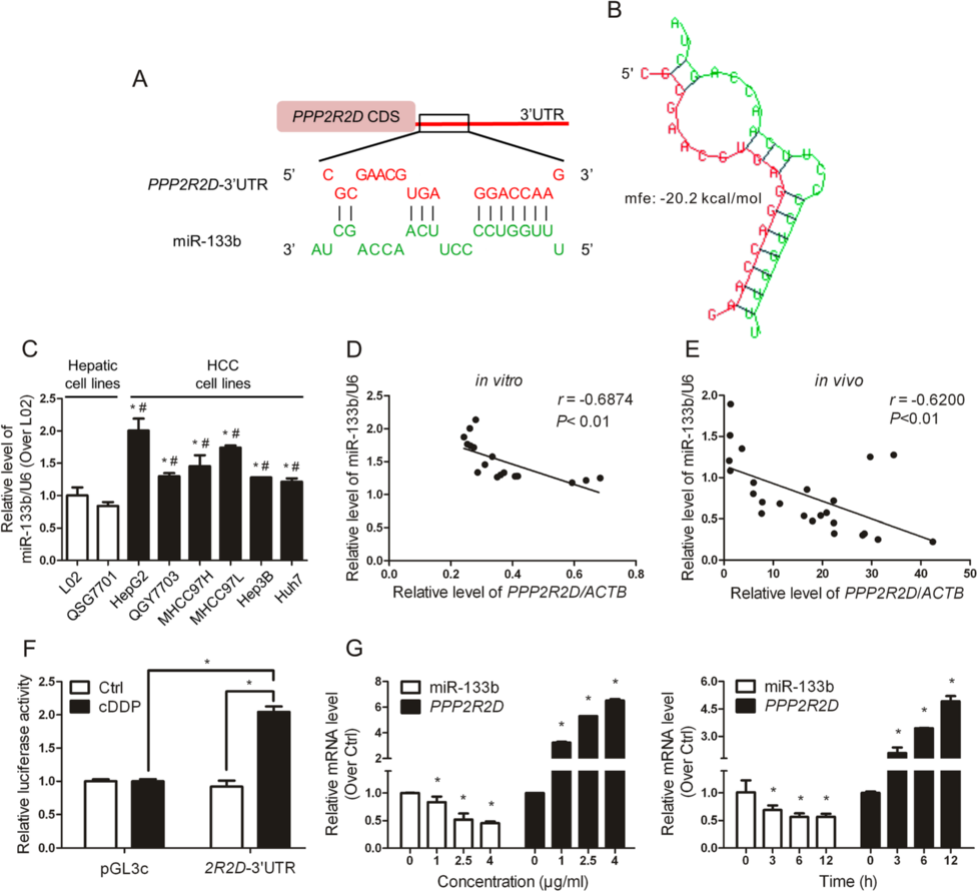
*PPP2R2D* is a direct downstream target gene of miR-133b. **a** The schematic diagram shows the *PPP2R2D* 3’UTR (*2R2D*-3’UTR) regions containing the binding site for miR-133b. **b** Predicted structure and free energy of binding between miR-133b (green) and *2R2D*-3’UTR (red). **c** The miR-133b expression in normal hepatic cell lines and HCC cell lines. *P < 0.01 as compared with L02 cells; ^#^ *P* < 0.01 as compared with QSG7701 cells. **d** Pearson’s correlation analysis of miR-133b and *PPP2R2D* mRNA levels among HCC cell lines. **e** Spearman’s correlation analysis of miR-133b and *PPP2R2D* mRNA levels in HCC xenograft tumors. **f** Luciferase activity of pGL3c-*2R2D*-3’UTR reporter gene in HepG2 cells treated with or without 2.5 μg/ml cDDP for 6 h. The data shown are the mean ± SD of three parallel samples, *P < 0.01. **g** miR-133b and *PPP2R2D* mRNA levels in HepG2 cells treated with 2.5 μg/ml cDDP for 0, 3, 6, or 12 h or treated with 0, 1, 2.5, or 4 μg/ml cDDP for 12 h. *P < 0.01 as compared with cDDP-untreated group (Ctrl)

In order to explore the epigenetic mechanisms of B55δ up-regulation induced by cDDP, microRNA.org and TargetScan databases were used in combination to screen for miRNAs which might target *PPP2R2D*. As shown in Fig. 6a, miR-133b was found to bind to the 3’UTR of *PPP2R2D*. The predicted free energy of binding was -20.2 kcal/mol (Fig. 6b). Furthermore, the expression level of miR-133b was significantly up-regulated in all HCC cell lines compared with normal hepatic cell lines (Fig. 6c), and it was inversely related to the expression of *PPP2R2D* shown in Fig. 1c. Correlation analyses showed that the expression of miR-133b was negatively correlated with *PPP2R2D* and B55δ *in vitro* and *in vivo* (Fig. 6d–e, Additional file 4: Figure S3). Based on these data, we hypothesized that decreased miR-133b might take part in cDDP-induced up-regulation of B55δ by targeting its mRNA transcripts. To confirm this hypothesis, a luciferase reporter assay was employed. The results revealed that cDDP increased the luciferase activity of the reporter construct bearing the *PPP2R2D* 3’UTR (Fig. 6f). Moreover, there was an apparent depletion of miR-133b, with a parallel elevation of *PPP2R2D*, induced by cDDP in a concentration- and time-dependent manner in HepG2 cells (Fig. 6g). In summary, cDDP might up-regulate *PPP2R2D* through decreasing the promotion of its degradation or the inhibition of its translation by miR-133b acting on the 3’UTR.

### miR-133b participates in cell cycle regulation by targeting *PPP2R2D*

**Fig. 7:**
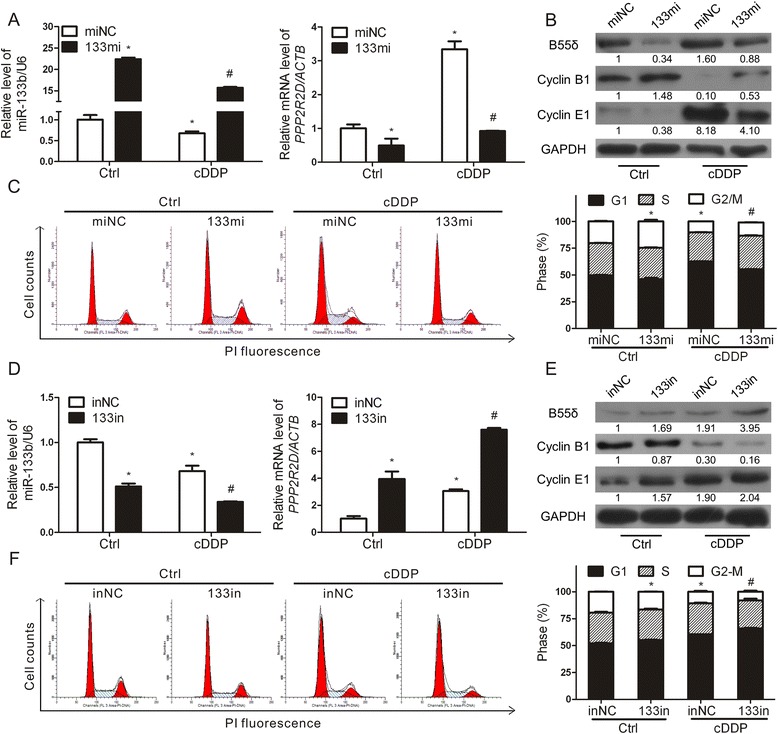
miR-133b participates in cell cycle regulation by targeting *PPP2R2D*. **a**-**c** After transfection with miNC or 133mi, HepG2 cells were treated with or without 2.5 μg/ml cDDP for 12 h. **a** miR-133b and *PPP2R2D* mRNA levels. **b** Protein levels of B55δ, Cyclin B1, and Cyclin E1. **c** Cell cycle distribution was analyzed by FCM. **d**-**f** HepG2 cells transfected with inNC or 133in were treated with or without cDDP. **d** qRT-PCR, **e** WB, and (**f**) FCM were conducted as before. The data are expressed as mean ± SD of three independent experiments. *P < 0.01 as compared with Ctrl group of miNC- or inNC-transfected HepG2 cells; ^#^ *P* < 0.01 as compared with cDDP group of miNC- or inNC-transfected HepG2 cells

To further verify the regulatory mechanism of miR-133b towards *PPP2R2D*, HepG2 cells were transfected with mimic negative control (miNC) or miR-133b mimic (133mi). The models were verified by qRT-PCR and WB. In response to 133mi, the levels of *PPP2R2D* mRNA and B55δ protein, not of the other PP2A subunits, were effectively suppressed (Additional file 5: Figure S4A–B, “Ctrl” in Fig. 7a–b). Moreover, 133mi reversed the up-regulation of *PPP2R2D* and B55δ induced by cDDP, and rescued G1 phase arrest (Fig. 7a–c). Also, inhibitor negative control (inNC) or miR-133b inhibitor (133in) was given to further confirm our results shown above. 133in enhanced the expression of *PPP2R2D* and B55δ, and accelerated G1 phase arrest (Additional file 5: Figure S4C–D, Fig. 7d–f). As a whole, our findings provided a potential molecular mechanism leading to the up-regulation of B55δ by cDDP chemotherapy, involving the decrease of miR-133b.

## Discussion

HCC is one of the most lethal forms of cancer in the world [18]. In recent years, molecule-targeted drugs, such as Sorafenib and Brivanib, have been developed for the treatment of liver cancer [19]. However, there are few drugs designed for HCC and most targeted therapeutic drugs are less effective [20]. cDDP is an old drug that is commonly used as a chemotherapeutic agent against advanced HCC [12]. However, the sensitivity to cDDP varies among HCC patients. Thus, the identification of effective targets for enhancing chemotherapeutic sensitivity is urgently required.

As a key tumor suppressor, PP2A has emerged as a novel target in alternative therapeutic strategies in many cancers [21–23]. Chien et al. reported that PP2A activation is crucial for deceleration of pancreatic tumorigenesis [24]. Furthermore, PP2A activation shows a promising anti-tumor effect in breast cancer [25]. Our previous studies have shown that the genetic variant -241 (-/G) (rs11453459) in the 5’-flanking region of *PPP2R1A* contributes to the decreased risk of HCC in a southern Chinese Han population [26]. Moreover, other studies have demonstrated that the regulation of regulatory B subunits of PP2A (PP2A-Bs), such as B55β and B56γ, play important roles in cancer development or treatment [27]. In the current study, *PPP2R2D* expression was found to be down-regulated in both HCC tumors and HCC cell lines. The difference in *PPP2R2D* expression between normal and tumor cells suggested it as a potential biomarker for HCC. Following treatment with cDDP, B55δ was increased. We hypothesize that increased B55δ enhances the sensitivity of HCC to cDDP chemotherapy. The current study conducted in stable *PPP2R2D*-knockdown and -overexpression cell lines validated this conclusion. The results showed that knockdown of B55δ markedly decreased the effect of cDDP, while overexpression of B55δ promoted the chemosensitivity of HepG2 cells and that of HCC xenograft tumors*.* Thus, our study has shown for the first time that PP2A holoenzyme containing the B55δ subunit is a potential molecular target which might enhance chemotherapy sensitivity of HCC. Further exploitation of B55δ might help patients overcome insensitivity of anti-cancer agents and advance the development of tailor-made treatments for HCC.

PP2A-B55δ is critical for the control of the entry into and exit from mitosis. On one hand, overexpression of B55δ blocks CDK1 activation and delays mitotic progression; on the other hand, depletion of B55δ accelerates entry into mitosis [28]. As an essential phosphatase, PP2A appears to act as a central modulator of phosphorylation to generate interphase and mitosis [29]. In our study, when B55δ was knocked down, CDK1 was partially increased, which reversed the G2/M reduction and G1 arrest induced by cDDP. In contrast, overexpression of B55δ held HepG2 cells in G1 phase by the phosphorylation of CDK1, increasing cDDP’s suppression of cell migratory ability, self-renewal capacity, and proliferation. The activation of PP2A plays a key initiating role in various pathways that lead to apoptosis in cancer cells [30, 31]. We also observed that overexpression of B55δ in the presence of cDDP resulted in down-regulation of the anti-apoptotic molecule Bcl-2 and up-regulation of pro-apoptotic molecules Bax and cleaved Caspase-3. Moreover, it was noteworthy that B55δ inhibited tumor growth while reducing the expression of the proliferation-related protein PCNA. These observations shed light on the underlying molecular mechanism of B55δ in the regulation of the cell cycle, and confirm the pivotal role that B55δ plays in increasing the sensitivity to chemotherapy in HCC.

Our previous studies showed that the transcriptional activities of *PPP2R2D* and *PPP2R1A* were regulated by the polymorphism and methylation through genetic and epigenetic mechanisms [26, 32]. To further investigate the miRNA-mediated epigenetic mechanisms of *PPP2R2D* up-regulation induced by cDDP, various approaches, including *in silico* analyses of putative mRNA/miRNA complexes, were applied. We confirmed for the first time that miR-133b regulates *PPP2R2D* post-transcriptional expression and translation by binding to complementary sequences of the 3’UTR of *PPP2R2D* mRNA. It has been postulated that miRNAs can function as either tumor suppressors or oncogenes by regulating protein-coding genes [33, 34]. miR-133b has been revealed to be down-regulated in Parkinson disease, human non-small cell lung, bladder, gastric, and colorectal cancers [35–39]. In this study, miR-133b was up-regulated in HCC cell lines, with a corresponding down-regulation of *PPP2R2D*. Thus, we speculate miR-133b might act as an oncogenic miRNA (oncomiR) in HCC. Recently, miRNAs have been considered as good biomarkers for early diagnosis and therapy of HCC [33]. Inhibition of oncomiRs has been introduced as a novel therapeutic strategy for cancer treatment [40]. By mimicking or inhibiting miR-133b, our study elucidated the miR-133b/*PPP2R2D* signaling pathway involved in cDDP chemotherapy. Taken together, these results suggest that miR-133b may serve as a gene-specific biomarker for estimating the prognosis of HCC. It would be of great interest to further investigate the functional characterization and regulatory mechanism of miR-133b and its derepression of PP2A-B55δ synthesis and function in the chemotherapy of HCC in both animal models and clinical samples.

## Conclusions

**Fig. 8:**
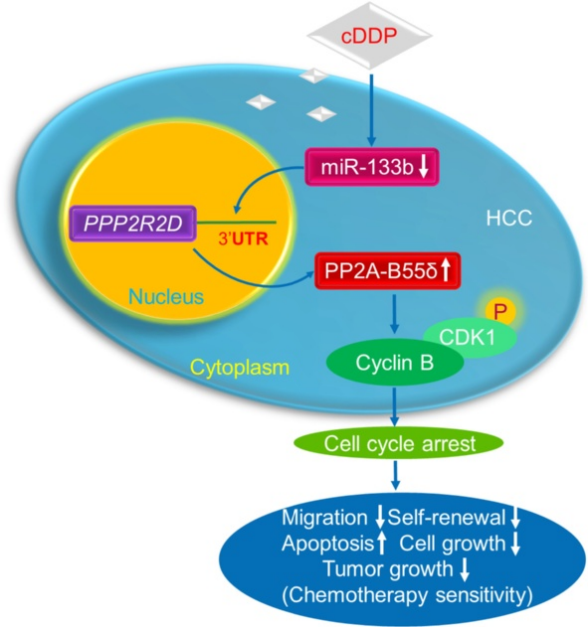
Schematic representation depicting the regulation of PP2A-B55δ by miR-133b and its role in promoting chemotherapy sensitivity of HCC

In summary, our current studies offer a new insight into the underlying mechanism of chemotherapy sensitivity of HCC and a possible target for intervention. PP2A-B55δ, under the regulation of miR-133b, modulates cell cycle progression by counteracting the activation of CDK1, and influences cell migration, colony formation, apoptosis, and proliferation both *in vitro* and *in vivo*, thus affecting the therapeutic response to cDDP (Fig. 8). Consequently, our findings suggest that PP2A-B55δ might be a potential therapeutic target for increasing the sensitivity of HCC to chemotherapy and that inhibition regulation of miR-133b may be an important aspect of using this target.

## Additional files

Additional file 1: Table S1. Primers used in this study. (DOC 54 kb) Additional file 2: Figure S1. The stable *PPP2R2D*-knockdown and -overexpression cell lines were verified by qRT-PCR and WB. The mRNA levels of a panel of PP2A subunits: *PPP2R1A*, *PPP2CA*, *PPP2R2A*, *PPP2R2D*, and *PPP2R5C* were detected in A HepG2-sh*GFP* and HepG2-sh*2R2D* cells, and C HepG2-pBabe and HepG2-*2R2Dc* cells (**P* < 0.01 as compared with HepG2-sh*GFP* or HepG2-pBabe cells). The protein levels of PP2A subunits (PP2A-Aα, -C, -B55α, -B55δ, and -B56γ) were measured in B HepG2-sh*GFP* and HepG2-sh*2R2D* cells, and D HepG2-pBabe and HepG2-*2R2Dc* cells. (TIF 1211 kb) Additional file 3: Figure S2. Quantitative immunohistochemical assessment of protein levels in xenograft tumors with or without cDDP treatment. The histogram shows the amounts of B55δ, Cyclin B1, Cyclin E1, and PCNA proteins determined by IPP 6.0 analysis of the micrographs of Fig. 5d. **P* < 0.01 as compared with Ctrl group of HepG2-pBabe cells. ^#^ *P* < 0.01 as compared with cDDP group of HepG2-pBabe cells. (TIF 528 kb) Additional file 4: Figure S3. Correlation analyses of miR-133b and B55δ protein expression *in vitro* and *in vivo*. A Pearson’s correlation analysis of miR-133b and B55δ among HCC cell lines. B Spearman’s correlation analysis of miR-133b and B55δ in HCC xenograft tumors. (TIF 450 kb) Additional file 5: Figure S4. The miR-133b mimic or inhibitor models were verified by qRT-PCR and WB. The mRNA levels of PP2A subunits: *PPP2R1A*, *PPP2CA*, *PPP2R2A*, *PPP2R2D*, and *PPP2R5C* were detected in HepG2 cells A transfected with miNC or 133mi, or C transfected with inNC or 133in (**P* < 0.01 as compared with miNC- or inNC-transfected HepG2 cells). The protein levels of PP2A subunits (PP2A-Aα, -C, -B55α, -B55δ, and -B56γ) were measured in HepG2 cells B transfected with miNC or 133mi, or D transfected with inNC or 133in. (TIF 1102 kb)

## Abbreviations

133in: miR-133b inhibitor
133mi: miR-133b mimic
*2R2Dc*: *PPP2R2D* coding sequence
3’UTR: 3’-untranslated region
B55δ: B55 subunit δ isoform
Bax: Bcl-2-associated X protein
Bcl-2: B-cell lymphoma 2-related protein
cDDP: cisplatin
CDK1: cyclin-dependent kinase 1
DAPI: 4, 6-diamidino-2-phenylindole
FCM: flow cytometry
GAPDH: glyceraldehyde-3-phosphate dehydrogenase
GEO: gene expression omnibus
GI: growth inhibition
HCC: hepatocellular carcinoma
IHC: immunohistochemistry
inNC: inhibitor negative control
miNC: mimic negative control
miR-133b: microRNA-133b
miRNAs: microRNAs
MTT: 3-(4, 5-dimethylthiazol-2-yl)-2, 5-diphenyltetrazolium bromide
OD: optical density
oncomiR: oncogenic miRNA
pBabe: plasmid pBabe-puro
p-CDK1: phosphorylated cyclin dependent kinase 1
PCNA: proliferating cell nuclear antigen
pGL3c: pGL3-control luciferase reporter vector
PI: propidium iodide
PP2A: protein phosphatase 2A
*PPP2R2D*: gene encoding PP2A-B55δ
qRT-PCR: quantitative real-time polymerase chain reaction
SD: standard deviation
sh*2R2D*: shRNA targeting the *PPP2R2D* mRNA sequence
sh*GFP*: shRNA targeting green fluorescent protein
shRNA: short hairpin RNA
WB: western blotting

## References

[1] Torre LA, Bray F, Siegel RL, Ferlay J, Lortet-Tieulent J, Jemal A (2015). Global cancer statistics, 2012. CA Cancer J Clin.

[2] Kulik LM, Chokechanachaisakul A (2015). Evaluation and management of hepatocellular carcinoma. Clin Liver Dis.

[3] Janssens V, Goris J, Van Hoof C (2005). PP2A: the expected tumor suppressor. Curr Opin Genet Dev.

[4] Seshacharyulu P, Pandey P, Datta K, Batra SK (2013). Phosphatase: PP2A structural importance, regulation and its aberrant expression in cancer. Cancer Lett.

[5] Jeong AL, Yang Y (2013). PP2A function toward mitotic kinases and substrates during the cell cycle. BMB Rep.

[6] Afonso-Grunz F, Muller S (2015). Principles of miRNA-mRNA interactions: beyond sequence complementarity. Cell Mol Life Sci.

[7] Marrone AK, Beland FA, Pogribny IP (2015). The role for microRNAs in drug toxicity and in safety assessment. Expert Opin Drug Metab Toxicol.

[8] Inui M, Martello G, Piccolo S (2010). MicroRNA control of signal transduction. Nat Rev Mol Cell Biol.

[9] Rottiers V, Naar AM (2012). MicroRNAs in metabolism and metabolic disorders. Nat Rev Mol Cell Biol.

[10] Gurtner A, Falcone E, Garibaldi F, Piaggio G (2016). Dysregulation of microRNA biogenesis in cancer: the impact of mutant p53 on Drosha complex activity. J Exp Clin Cancer Res.

[11] Ruvolo PP (2015). The Interplay between PP2A and microRNAs in Leukemia. Front Oncol.

[12] Ishikawa T (2009). Future perspectives on the treatment of hepatocellular carcinoma with cisplatin. World J Hepatol.

[13] Liao K, Xia B, Zhuang QY, Hou MJ, Zhang YJ, Luo B (2015). Parthenolide inhibits cancer stem-like side population of nasopharyngeal carcinoma cells via suppression of the NF-κB/COX-2 pathway. Theranostics.

[14] Udali S, Guarini P, Ruzzenente A, Ferrarini A, Guglielmi A, Lotto V (2015). DNA methylation and gene expression profiles show novel regulatory pathways in hepatocellular carcinoma. Clin Epigenetics.

[15] Wurmbach E, Chen YB, Khitrov G, Zhang W, Roayaie S, Schwartz M (2007). Genome-wide molecular profiles of HCV-induced dysplasia and hepatocellular carcinoma. Hepatology.

[16] Villa E, Critelli R, Lei B, Marzocchi G, Camma C, Giannelli G (2015). Neoangiogenesis-related genes are hallmarks of fast-growing hepatocellular carcinomas and worst survival. Results from a prospective study. Gut.

[17] Kelland L (2007). The resurgence of platinum-based cancer chemotherapy. Nat Rev Cancer.

[18] Dhanasekaran R, Limaye A, Cabrera R (2012). Hepatocellular carcinoma: current trends in worldwide epidemiology, risk factors, diagnosis, and therapeutics. Hepat Med.

[19] Llovet JM, Villanueva A, Lachenmayer A, Finn RS (2015). Advances in targeted therapies for hepatocellular carcinoma in the genomic era. Nat Rev Clin Oncol.

[20] Chen C, Wang G (2015). Mechanisms of hepatocellular carcinoma and challenges and opportunities for molecular targeted therapy. World J Hepatol.

[21] Cristobal I, Manso R, Rincon R, Carames C, Senin C, Borrero A (2014). PP2A inhibition is a common event in colorectal cancer and its restoration using FTY720 shows promising therapeutic potential. Mol Cancer Ther.

[22] Sangodkar J, Mazhar S, Kastrinsky D, Ohlmeyer M, Narla G (2014). Development of small molecule activators of protein phosphatase 2A for the treatment of lung cancer. Eur J Cancer.

[23] Perrotti D, Neviani P (2013). Protein phosphatase 2A: a target for anticancer therapy. Lancet Oncol.

[24] Chien WW, Sun QY, Lee KL, Ding LW, Wuensche P, Torres-Fernandez LA (2015). Activation of protein phosphatase 2A tumor suppressor as potential treatment of pancreatic cancer. Mol Oncol.

[25] Rincon R, Cristobal I, Zazo S, Arpi O, Menendez S, Manso R (2015). PP2A inhibition determines poor outcome and doxorubicin resistance in early breast cancer and its activation shows promising therapeutic effects. Oncotarget.

[26] Chen HF, Mai JR, Wan JX, Gao YF, Lin LN, Wang SZ (2013). Role of a novel functional variant in the *PPP2R1A* promoter on the regulation of PP2A-Aalpha and the risk of hepatocellular carcinoma. PLoS ONE.

[27] Chen W, Wang Z, Jiang C, Ding Y (2013). PP2A-mediated anticancer therapy. Gastroenterol Res Pract.

[28] Mochida S, Ikeo S, Gannon J, Hunt T (2009). Regulated activity of PP2A-B55δ is crucial for controlling entry into and exit from mitosis in *Xenopus* egg extracts. EMBO J.

[29] Krasinska L, Domingo-Sananes MR, Kapuy O, Parisis N, Harker B, Moorhead G (2011). Protein phosphatase 2A controls the order and dynamics of cell-cycle transitions. Mol Cell.

[30] Guichard C, Pedruzzi E, Fay M, Marie JC, Braut-Boucher F, Daniel F (2006). Dihydroxyphenylethanol induces apoptosis by activating serine/threonine protein phosphatase PP2A and promotes the endoplasmic reticulum stress response in human colon carcinoma cells. Carcinogenesis.

[31] Garcia A, Cayla X, Guergnon J, Dessauge F, Hospital V, Rebollo MP (2003). Serine/threonine protein phosphatases PP1 and PP2A are key players in apoptosis. Biochimie.

[32] Chen HF, Lin LN, Chen YX, Wan JX, Luo J, Zhang CZ (2012). Identification and functional analysis of variant haplotypes in the 5’-flanking region of protein phosphatase 2A-Bδ gene. PLoS ONE.

[33] Yang N, Ekanem NR, Sakyi CA, Ray SD (2015). Hepatocellular carcinoma and microRNA: new perspectives on therapeutics and diagnostics. Adv Drug Deliv Rev.

[34] Cheng Z, Wang HZ, Li XT, Wu ZW, Han Y, Li YY (2015). MicroRNA-184 inhibits cell proliferation and invasion, and specifically targets TNFAIP2 in Glioma. J Exp Clin Cancer Res.

[35] Zhao N, Jin LR, Fei GQ, Zheng ZY, Zhong CJ (2014). Serum microRNA-133b is associated with low ceruloplasmin levels in Parkinson’s disease. Parkinsonism Relat Disord.

[36] Liu LX, Shao XY, Gao W, Zhang Z, Liu P, Wang RS (2012). MicroRNA-133b inhibits the growth of non-small-cell lung cancer by targeting the epidermal growth factor receptor. FEBS J.

[37] Zhou YF, Wu DY, Tao J, Qu P, Zhou ZD, Hou JQ (2013). MicroRNA-133 inhibits cell proliferation, migration and invasion by targeting epidermal growth factor receptor and its downstream effector proteins in bladder cancer. Scand J Urol.

[38] Guo L, Bai H, Zou D, Hong T, Liu J, Huang J (2014). The role of microRNA-133b and its target gene FSCN1 in gastric cancer. J Exp Clin Cancer Res.

[39] Akcakaya P, Ekelund S, Kolosenko I, Caramuta S, Ozata DM, Xie H (2011). miR-185 and miR-133b deregulation is associated with overall survival and metastasis in colorectal cancer. Int J Oncol.

[40] Cheng CJ, Bahal R, Babar IA, Pincus Z, Barrera F, Liu C (2015). MicroRNA silencing for cancer therapy targeted to the tumour microenvironment. Nature.

