# Supplementary material for: Protein phosphatase 2A-B55δ enhances chemotherapy sensitivity of human hepatocellular carcinoma under the regulation of microRNA-133b
Source: J Exp Clin Cancer Res. 2016 Apr 14;35:67. doi: 10.1186/s13046-016-0341-z (PMC4831140; doi:10.1186/s13046-016-0341-z)
Supplement: Additional file 1: Table S1. — Primers used in this study. (DOC 54 kb) [file 13046_2016_341_MOESM1_ESM.doc]

**Additional file 1: Table S1.** Primers used in this study.

| **Designation** | **Primer Sequence** | **Use** |
| --- | --- | --- |
| *PPP2R2Dc*+89PF | 5'- CGT GAA CAA GAG AAT AAA AGC CG -3' | qRT-PCR |
| *PPP2R2Dc*+394PR | 5'- CTT CAA TAT TGG GAC CCG TAG -3' | qRT-PCR |
| *ACTB* PF | 5'- CAC CAG GGC GTG ATG GT -3' | qRT-PCR |
| *ACTB* PR | 5'- CTC AAA CAT GAT CTG GGT CAT -3' | qRT-PCR |
| *PPP2R1Ac*+1667PF | 5'- TCT GCA TCA ATG TGC TGT CT -3' | qRT-PCR |
| *PPP2R1Ac*+1836PR | 5'- TTC ACT CTG CAA GGT GCT GT -3' | qRT-PCR |
| *PPP2CAc*+1125PF | 5'-CTA GTG ATG GAG GGA TAT AAC T -3' | qRT-PCR |
| *PPP2CAc*+1267PR | 5'-TCA AAC TGC AAG AAA GAG TAT T -3' | qRT-PCR |
| *PPP2R2Ac*+745PF | 5'- CAG TTA CTA CAC TAC GAG TGC -3' | qRT-PCR |
| *PPP2R2Ac*+948PR | 5'-CCA CAA TGT TAA AAC TCC TGT C -3' | qRT-PCR |
| *PPP2R5Cc*+1419PF | 5'- CAA AGC CAA TCC CCA GTA C -3' | qRT-PCR |
| *PPP2R5Cc*+1567PR | 5'-TCG GAT CTT TCT GTG CCT GA -3' | qRT-PCR |
| sh*2R2D+*1899AgePF | 5'- CCG GGC ATA GTT AAG CCG GAC ATT TCT CGA GAA ATG TCC GGC TTA ACT ATG CTT TTT G -3' | pLKO.1-sh*2R2D* construct |
| sh*2R2D+*1899EcoPR | 5'- AAT TCA AAA AGC ATA GTT AAG CCG GAC ATT TCT CGA GAA ATG TCC GGC TTA ACT ATG C -3' | pLKO.1-sh*2R2D* construct |
| sh*2R2D+*1823AgePF | 5'- CCG GGC TGC CAC CAA TAA CTT GTA CCT CGA GGT ACA AGT TAT TGG TGG CAG CTT TTT G -3' | pLKO.1-sh*2R2D* construct |
| sh*2R2D+*1823EcoPR | 5'- AAT TCA AAA AGC TGC CAC CAA TAA CTT GTA CCT CGA GGT ACA AGT TAT TGG TGG CAG C -3' | pLKO.1-sh*2R2D* construct |
| sh*2R2D+*1358AgePF | 5'- CCG GGT CCT TCT TCT CAG AAA TAA TCT CGA GAT TAT TTC TGA GAA GAA GGA CTT TTT G -3' | pLKO.1-sh*2R2D* construct |
| sh*2R2D+*1358EcoPR | 5'- AAT TCA AAA AGT CCT TCT TCT CAG AAA TAA TCT CGA GAT TAT TTC TGA GAA GAA GGA C -3' | pLKO.1-sh*2R2D* construct |
| sh*2R2D+*747AgePF | 5'- CCG GGC ACC TTT CAA AGT CAT GAA CCT CGA GGT TCA TGA CTT TGA AAG GTG CTT TTT G -3' | pLKO.1-sh*2R2D* construct |
| sh*2R2D+*747EcoPR | 5'- AAT TCA AAA AGC ACC TTT CAA AGT CAT GAA CCT CGA GGT TCA TGA CTT TGA AAG GTG C -3' | pLKO.1-sh*2R2D* construct |
| sh*GFP* PF | 5'- CCG GGC AAG CTG ACC CTG AAG TTC ATC TCG AGA TAC GGC TGT CCA TAA ACT GGT TTT TG -3' | pLKO.1-sh*GFP* construct |
| sh*GFP* PR | 5'- AAT TCA AAA AGC AAG CTG ACC CTG AAG TTC ATC TCG AGA TAC GGC TGT CCA TAA ACT GG -3' | pLKO.1-sh*GFP* construct |
| *2R2Dc*+506PF | 5'-ATG GCA GGA GCC GGA -3' | pBabe-*2R2Dc* construct |
| *2R2Dc*+1871PR | 5'- GTC TCT AGT TGA TTT TGT CCT GGA -3' | pBabe-*2R2Dc* construct |
| *2R2Dc*+506BamFlagPF | 5'-GAA TGG ATC CAT GCT TAT CGT CGT CAT CCT TGT AAT CGC AGG AGC CGG A -3' | pBabe-*2R2Dc* construct |
| *2R2Dc*+1867SalPR | 5'- GAT TGT CGA CCT AGT TGA TTT TGT CC -3' | pBabe-*2R2Dc* construct |
| *2R2D*-3’UTR+1868EcoPF | 5'- GTA TGA ATT CAG ACG CGA ACG TGA GGA -3' | pGL3c-*2R2D*-3’UTR construct |
| *2R2D*-3’UTR+2421XbaPR | 5'- GAA TTC TAG ATT TAT TGA GCG CTC AC -3' | pGL3c-*2R2D*-3’UTR construct |

PF, forward primer; PR, reverse primer; Age, Age I; Eco, EcoR I; Bam, BamH I; Sal, Sal I; Xba, Xba I.
