# Supplementary figures and images for: Protein phosphatase 2A-B55δ enhances chemotherapy sensitivity of human hepatocellular carcinoma under the regulation of microRNA-133b
Source: J Exp Clin Cancer Res. 2016 Apr 14;35:67. doi: 10.1186/s13046-016-0341-z (PMC4831140; doi:10.1186/s13046-016-0341-z)

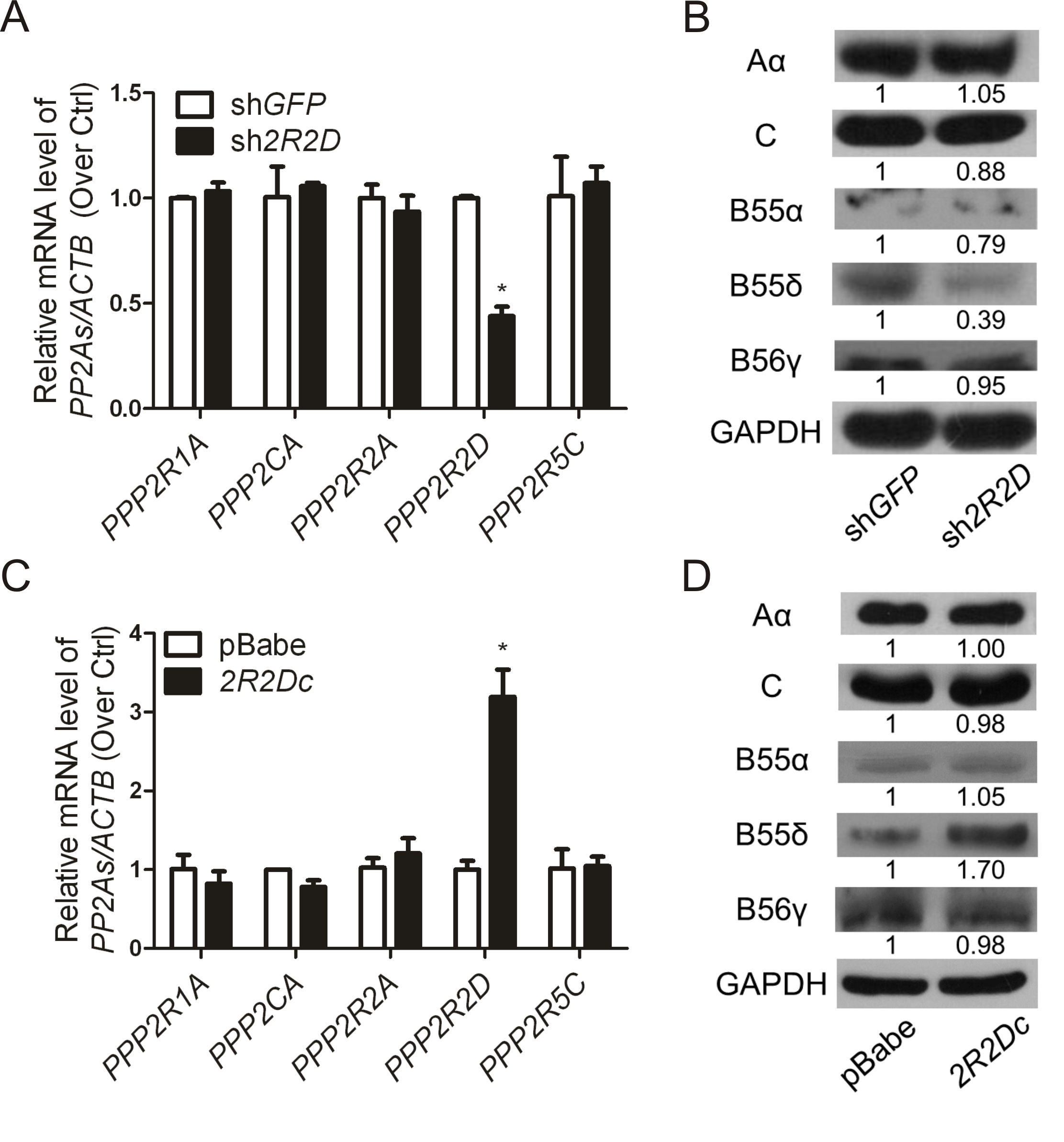

Supplement: Additional file 2: Figure S1. — The stable PPP2R2D-knockdown and -overexpression cell lines were verified by qRT-PCR and WB. The mRNA levels of a panel of PP2A subunits: PPP2R1A, PPP2CA, PPP2R2A, PPP2R2D, and PPP2R5C were detected in A HepG2-shGFP and HepG2-sh2R2D cells, and C HepG2-pBabe and HepG2-2R2Dc cells (*P < 0.01 as compared with HepG2-shGFP or HepG2-pBabe cells). The protein levels of PP2A subunits (PP2A-Aα, -C, -B55α, -B55δ, and -B56γ) were measured in B HepG2-shGFP and HepG2-sh2R2D cells, and D HepG2-pBabe and HepG2-2R2Dc cells. (TIF 1211 kb) [file 13046_2016_341_MOESM2_ESM.tif]

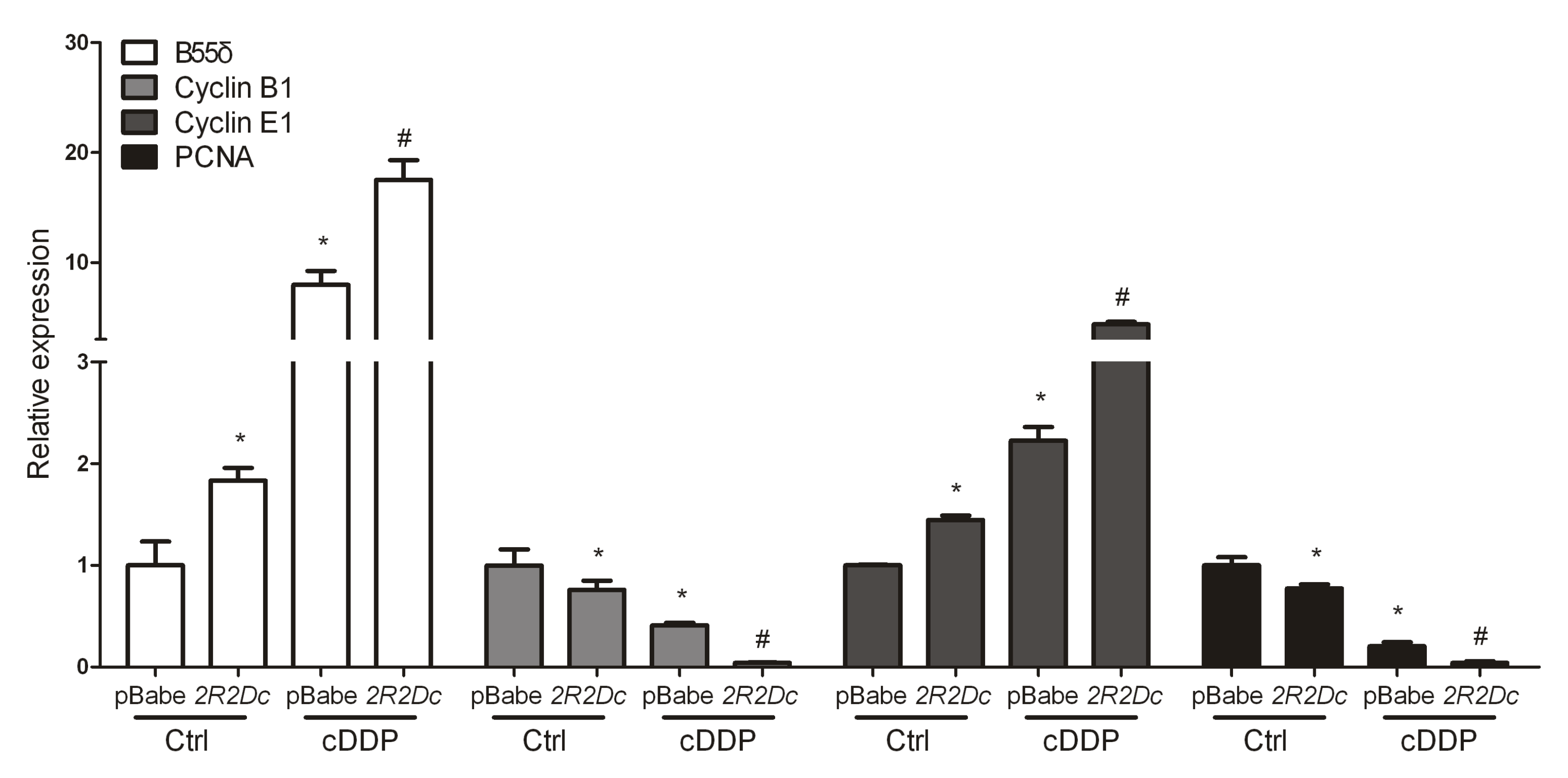

Supplement: Additional file 3: Figure S2. — Quantitative immunohistochemical assessment of protein levels in xenograft tumors with or without cDDP treatment. The histogram shows the amounts of B55δ, Cyclin B1, Cyclin E1, and PCNA proteins determined by IPP 6.0 analysis of the micrographs of Fig. 5d. *P < 0.01 as compared with Ctrl group of HepG2-pBabe cells. # P < 0.01 as compared with cDDP group of HepG2-pBabe cells. (TIF 528 kb) [file 13046_2016_341_MOESM3_ESM.tif]

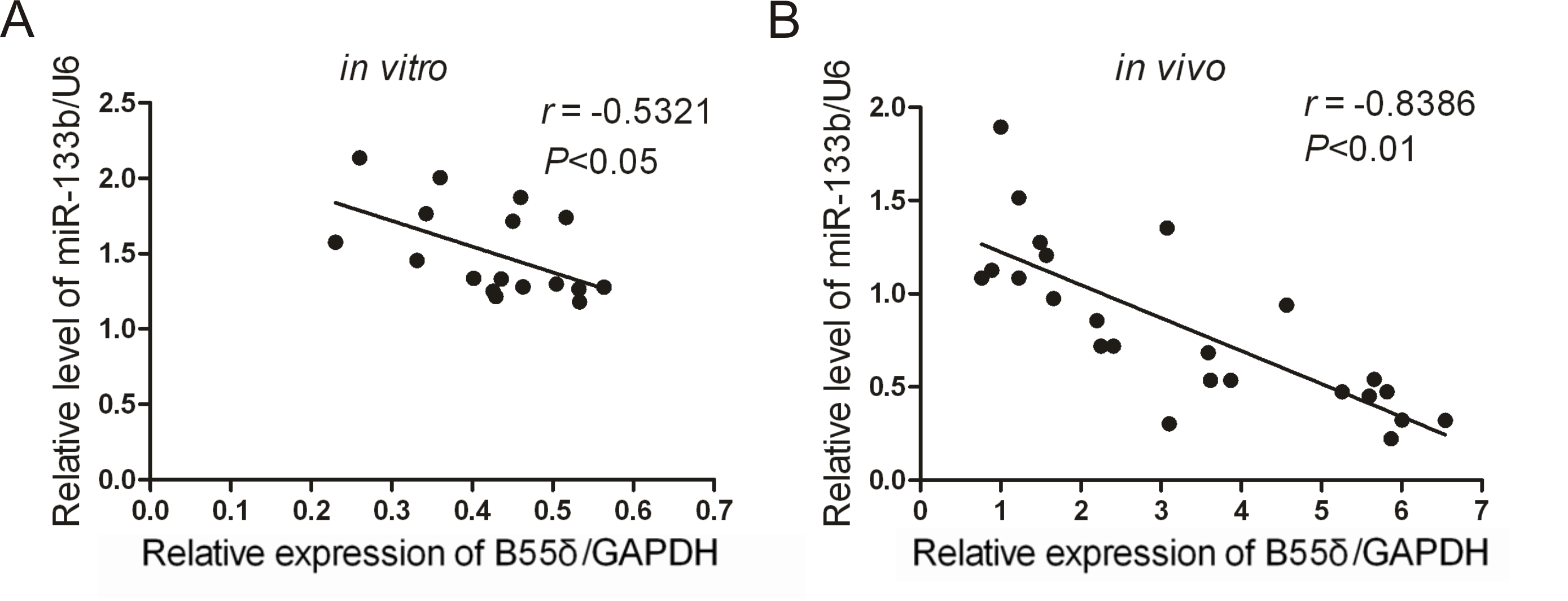

Supplement: Additional file 4: Figure S3. — Correlation analyses of miR-133b and B55δ protein expression in vitro and in vivo. A Pearson’s correlation analysis of miR-133b and B55δ among HCC cell lines. B Spearman’s correlation analysis of miR-133b and B55δ in HCC xenograft tumors. (TIF 450 kb) [file 13046_2016_341_MOESM4_ESM.tif]

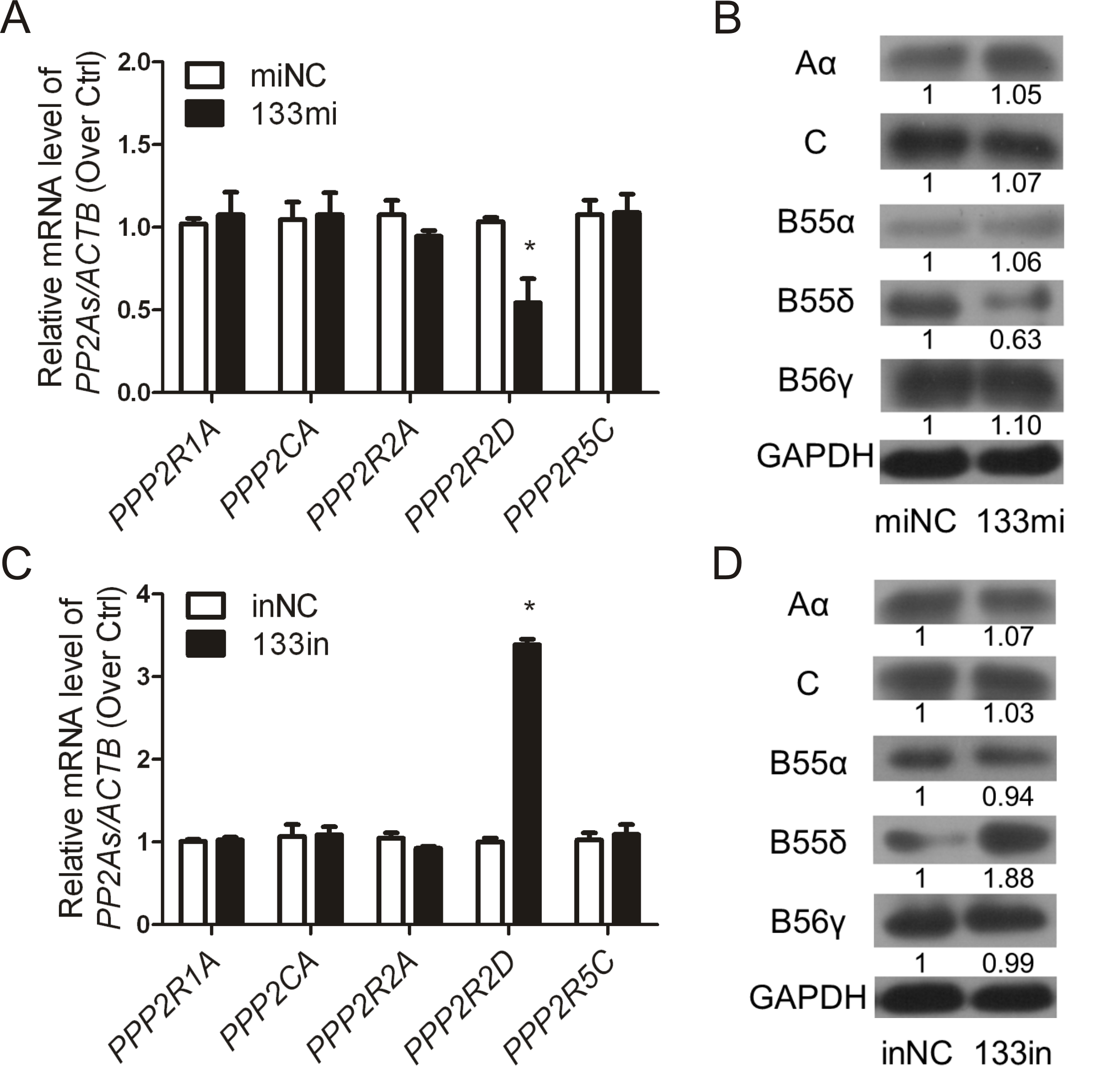

Supplement: Additional file 5: Figure S4. — The miR-133b mimic or inhibitor models were verified by qRT-PCR and WB. The mRNA levels of PP2A subunits: PPP2R1A, PPP2CA, PPP2R2A, PPP2R2D, and PPP2R5C were detected in HepG2 cells A transfected with miNC or 133mi, or C transfected with inNC or 133in (*P < 0.01 as compared with miNC- or inNC-transfected HepG2 cells). The protein levels of PP2A subunits (PP2A-Aα, -C, -B55α, -B55δ, and -B56γ) were measured in HepG2 cells B transfected with miNC or 133mi, or D transfected with inNC or 133in. (TIF 1102 kb) [file 13046_2016_341_MOESM5_ESM.tif]
